# An amplicon-based approach for full-genome characterization of HPV16

**DOI:** 10.1128/spectrum.03073-24

**Published:** 2025-06-09

**Authors:** Isabelle Malet, Inès Draa, Valentin Leducq, Fanny Vuong, Pascale Bonnafous, Anne-Geneviève Marcelin, Vincent Calvez, Aude Jary

**Affiliations:** 1 Sorbonne Université, INSERM, Institut Pierre Louis d’Epidémiologie et de Santé Publique, Assistance Publique—Hôpitaux de Paris (AP-HP), Hôpitaux Universitaires Pitié-Salpêtrière Charles-Foix, Laboratoire de Virologie, Paris, Île-de-France, France; University of Manitoba, Winnipeg, Manitoba, Canada

**Keywords:** HPV16, full genome, multiplex PCR, next-generation sequencing

## Abstract

Among the various genotypes of the human papillomavirus (HPV), the HPV16 genotype is the most oncogenic by far, and some (sub)lineages may be associated with an increased risk of cancer. Thus, characterizing the full genome is important to understand the link between the HPV16 genome variability and its transforming role. We set up a multiplex PCR approach combined with Oxford Nanopore Technologies for the HPV16 full-genome characterization. The primers were designed with PrimalScheme, and the optimization of the monoplex/multiplex PCR was performed on SiHa cells. After library preparation, the sequencing was performed on a GridION sequencing device to determine the sensitivity, the specificity, and the performance of the method. Fourteen primer pairs were selected to span the full genome. With the monoplex PCR, 12 primer pairs showed good amplification, while a double concentration of two primers was required to improve the amplification. We then performed a multiplex PCR approach by generating two pools comprising non-overlapping primer pairs. The multiplex PCR showed good sensitivity, allowing the amplification of HPV16 with a Ct value below 27 and providing a coverage greater than 99.9% and a sequencing depth greater than 100×. In addition, the specificity of the method was validated by the absence of amplification of other high-risk HPV genotypes compared with HPV16. We set up an amplicon-based approach to characterize the full genome and diversity of the HPV16 (sub)lineages. This approach shows good sensibility and specificity with limited cost, opening new perspectives in the field of whole-genome HPV sequencing.

**IMPORTANCE:**

HPV16 is by far the most oncogenic genotype, so characterizing the genetic variability of its genome is important to better understand the link with its transforming role. We developed an amplicon-based approach combined with Oxford Nanopore Technologies next-generation sequencing to overlap the HPV16 genome, which is easy to implement in the laboratory and inexpensive in the field.

## INTRODUCTION

Human papillomavirus (HPV) is the most common type of sexually transmitted infection (STI) in the world. Around 80% of people have been infected with it, but in most cases, the immune system will clear the infection without any disease (1, 2). If HPV infection persists, this virus can cause several cancers, including 100% of cervical cancers (SCC), 90% of anal cancers (ASCC), and 30% of vulva cancers (VSCC) (3). These cancers are preceded by pre-cancerous lesions from low-grade squamous intraepithelial lesions (i.e., CIN1, AIN1, or VIN1) to high-grade squamous intraepithelial lesions (i.e., CIN2 and CIN3, AIN2 and AIN3, VIN2 and VIN3), which develop over decades and can be prevented by regular screening and vaccination (4).

HPV is a non-enveloped virus with a circular double-stranded DNA surrounded by an icosahedral capsid. The DNA is around 7.9 kilobase pairs (kbp) in length, encoding for two structural capsid proteins, *Late 1* (L1) and *Late 2* (L2), and six *Early* proteins (E1, E2, E4, E5, E6, and E7) involved in the genome replication and the transcription/expression of viral proteins. The upstream regulatory region (URR) is a regulatory region, which also contains the origin of genome replication (5, 6). The oncoproteins E6 and E7 play a central role during HPV-mediated carcinogenesis by interacting with multiple proteins like the host cell tumor suppressors p53 and retinoblastoma protein (pRb) and disrupting many cellular processes (5, 6). According to the International Agency for Research on Cancer, 13 high-risk HPV (hrHPV) are currently classified as oncogenic (group 1) and probably oncogenic (group 2A) (7). Among them, the HPV16 genotype is the most carcinogenetic by far, involved in nearly 50% of SCC, 90% of ASCC, 68% of VSCC, and also about 82% of HPV-induced oropharyngeal squamous cell carcinoma (3, 8, 9). Among this genotype, lineages (1 to 10% differences) and sublineages (0.5 to 1% differences) have been defined at the whole-genome level (i.e., lineage A [A1–A4] mainly reported in Europe, lineages B [B1–B4] and C [C1–C4] mainly in Africa, and lineage D [D1–D4] mainly in Asia and America) (10). However, in cervical and anal cancers, certain sub-lineages have also been associated with an increased risk of (pre)cancerous lesions compared to A1/A2 sublineages (11, 12).

With the advancement of next-generation sequencing (NGS), viral genome determination has been drastically improved. However, it still could be challenging because of low DNA available, large viral genome, and the presence of contaminating host DNA, which we want to free ourselves from (13). In the case of HPV, which is the focus of this study, its relatively small genome of about 8,000 bp may make it easier to sequence compared to larger viral genomes. Enrichment approaches, such as probe capture or overlapping PCR panels (also called amplicon-based PCR or multiplex PCR), combined with NGS technologies, have clearly expanded the possibility of characterizing the entire viral genome (13–15). Additionally, the enrichment reduces the amount of sequence data needed to generate sufficient coverage of each genome, thereby allowing larger numbers of samples to be sequenced in multiplexes and reducing cost.

As HPV16 is the most important genotype in the carcinogenesis of HPV lesions, and its genome size is small, we set up a multiplex PCR approach combined with ONT sequencing to overlap its full genome with a low amount of starting DNA required.

## MATERIALS AND METHODS

### Samples

The setup of multiplex PCR before sequencing was performed on SiHa cells (ATCC: HTB-35), which is a cell line issued from a cervical carcinoma tissue positive for the HPV16 genotype. Each cell contains one to two integrated copies of the viral genome. The quantification of total DNA in the extract was estimated at 34.6 ng/µL. A range of SiHa cell line extract concentration from 1 to 8 ng DNA was first tested with primer pairs amplifying fragments #1 and #11, and the results of amplification are shown in Fig. S1. Except for 1 ng for which amplification was weak, good amplification was observed with the concentration of 2 ng and higher. For the next experiments, the concentration of 4 ng was used.

To assess the performance of our primers, two samples issued from patients tested positive for HPV16 in cervical scrape with low Ct values (P37, Ct = 21.3, P89, Ct = 21.4) were subjected to the full process of amplification and sequencing. The sensitivity of our method was determined by testing HPV16-positive samples with Ct values ranging from 19.3 to 36.8 and the specificity by testing samples positive for several other hrHPV genotypes.

### DNA extraction and HPV16 quantification

Briefly, 1 mL of cervical scrape was centrifuged; the supernatant was discarded; and the cell pellet was resuspended in 200 µL of phosphate buffered serum 1×. Then, DNA extraction was performed using the NucliSENS EasyMag total nucleic acid extractor (Biomérieux, Marcy l’Etoile, France) according to the manufacturer’s instructions. As part of the diagnosis, samples were tested for HPV detection using the Anyplex II HPV-HR Detection Kit (Seegene), which allows the semi-quantification of 14 hrHPV genotypes (+++: detection before 30 Ct, ++: detection between 30 and 40 Ct, +: detection between 40 and 50 Ct). For our study, HPV was tested again with the Allplex HPV28 Detection Kit (Seegene, Seoul, South Korea) to determine the Ct value of HPV16. A human gene is detected by these assays as an internal control to validate both sampling and PCR methods.

### HPV16 full-genome PCR setup

**Fig 1:**
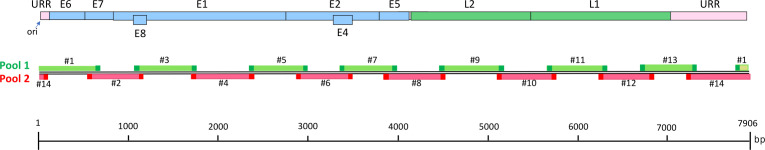
Schematic representation of the primer pair design with PrimalScheme and overlapping the full genome of HPV16. The first line represents HPV16 genes and genome, and the second line represents the fragments generated with primer pairs and their position on the genome (scale in base pairs). URR: upstream regulatory region; Ori: replication origin; bp: base pairs.

Primers were designed with PrimalScheme (https://primalscheme.com/) on a multiple alignment of HPV16 nucleotide reference genomes, including 41 strains from different lineages/sublineages (i.e., 17 lineage A [12 A1, 1 A3, and 4 A4], four lineage B, four lineage C, and 16 lineage D [1 D1, 6 D2, and 9 D3]) (Table S1) (10) to generate 14 fragments of about 700 bp overlapping by an average of 95 nucleotides across the entire genome (Fig. 1, Table S2). In a second step, primer pairs #1 and #14 were modified at the end and beginning of the genome so that the two fragments were overlapping in the episomal form of the HPV16 genome. Agarose gel electrophoresis was used to detect fragments after amplification.

The 14 primer pairs were evaluated on 4 ng of DNA from the SiHa cell line extract (in 2.5 µL) using PrimeSTAR GXL DNA polymerase (Takara Bio) according to the manufacturer’s instructions. The reaction mixture consisted of 0.5 µL PrimeSTAR GXL DNA polymerase (0.75 U), 5 µL 5× PrimeSTAR GXL buffer, 2 µL dNTP mix (final concentration 200 µM), 0.75 µL of each primer at 10 µM (final concentration 0.2 µM), and 13.5 µL water in a final volume of 25 µL. The PCR program was as follows: 40 cycles of 10 s at 98°C (denaturation), 15 s at 60°C (annealing), and 50 s at 68°C (elongation).

### Sanger sequencing

To check whether the fragments belonged to the HPV16 genome and spanned the full genome as well, Sanger sequencing was performed on the 14 amplified regions independently. Briefly, 2 µL of amplified DNA was subjected to bidirectional sequencing with BigDye Terminator Chemistry (Thermo Fisher Scientific) with an analysis of the reaction products on an ABI sequencer. The visualization of the final consensus sequence after alignment on the HPV16 reference genome (NC_001526.4) was performed on Geneious Prime 2022.2.2.

### Oxford Nanopore Technologies sequencing

Fragments covering the whole genome were sequenced on the Oxford Nanopore Technologies platform with a GridION instrument. Briefly, libraries were prepared using the Rapid Barcoding 96 Kit (SQK-RBK 110.96) and sequenced on Flow Cell R9.4.1 (FLO-MIN 106D). Resulting sequences were demultiplexed and base-called using Dorado (ONT) with the "Super Accurate" model. Sequences exceeding 200 bp with Q-scores above 10 were subsequently aligned to reference NC_001526.4 (National Center for Biotechnology Information GenBank) using Minimap2 (v2.26). Variant calling was carried out from these alignments using Medaka (v1.12.1). Consensus sequences were generated using BCFtools (v1.17), masking positions with coverage less than 25× using BEDtools (v2.31.1). The consensus sequences were used to produce a multi-alignment using MAFFT (v7.520) with several reference genomes associated with HPV16 sublineages A1, A2, A3, A4, B, C, D1, D2, and D3, as well as six genomes of other *Papillomaviridae* species serving as roots for the phylogenetic analysis. The multi-alignment was used to produce a maximum likelihood phylogenetic tree using IQ-TREE (v2.2.5) with the SH-aLRT test and 1,000 ultrafast bootstraps. The phylogenetic tree was visualized with iTOL (itol.embl.de).

### Statistical analysis

Continuous variables were expressed as median and interquartile (IQR) and categorical variables as number and percentage (%). *P* values were calculated using Mann-Whitney *U* test for quantitative variables and Fisher’s exact *t*-test for categorical variables. *P* value < 0.005 was considered significant.

## RESULTS

### Setup and optimization of the 14 pairs of primers on SiHa cells

#### Amplification

**Fig 2:**
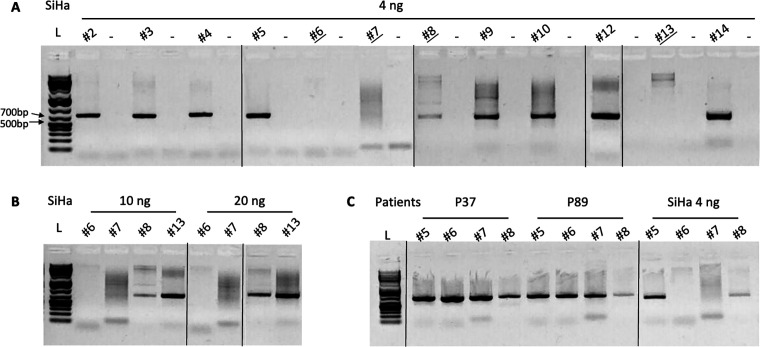
Agarose gel showing the amplification of the different fragments of HPV16 with different conditions. (**A**) Fourteen fragments with 4 ng of the SiHa cell line, (**B**) fragments #6, #7, #8, and #13 with 10 and 20 ng of the SiHa cell line, and (**C**) fragments #5, #6, #7, and #8 in patients P36 and P37 and in the SiHa cell line. L: ladder.

The 12 primer pairs (other than fragments #1 and #11) were tested and showed an overall good amplification of fragments of approximately 700 bp (Fig. 2A), except for fragment #8, which showed weak amplification, and fragments #6, #7, and #13, which showed no amplification (Fig. 2A).

To assess whether primer pairs #6, #7, #8, and #13 lacked sensitivity, 10 and 20 ng of DNA from the SiHa cell line extract were amplified using the same PCR conditions. Gel analysis showed that fragment #8 was still weakly amplified unlike fragments #13, which showed a correct amplification band, while fragments #6 and #7 remained non-amplifiable (Fig. 2B).

The failure of amplification with primer pairs #6 and #7 can be explained by the fact that these primers mapped in the E2 gene. Indeed, this gene is precisely disrupted due to the integration of the HPV genome into the host cell genome in the SiHa cell line (Fig. S2). We confirmed this by amplifying two longer fragments of about 1,500 bp using either the forward primer of pair #7 and the reverse primer of pair #8 (on each side of the supposed cleavage region) or the forward primer of pair #8 and the reverse primer of pair #9 (outside the supposed cleavage region). As expected, we only obtained an amplification band when the primers were located outside of the cleavage region (Fig. S3).

Finally, to validate primer pairs #6 and #7, they were tested on two patient samples (P37 and P89), which were positive for HPV16 but not associated with HPV lesions and thus expected to be without integration of the viral genome. The amplification of the two regions was correct, showing a fragment of 700 bp with an intense band (Fig. 2C).

#### Sanger sequencing

In total, the full genome of the SiHa cell line was generated by Sanger sequencing (excluding nucleotides from 3,073 to 3,947 corresponding to the two non-amplified regions #6 and #7), as well as two samples corresponding to patients P37 and P89 (including fragments #6 and #7). The three consensus sequences generated mapped to reference sequence NC_001526.4, confirming the specificity of the 14 fragments with respect to HPV16 (data not shown).

### Setup and optimization of the multiplex PCR approach on SiHa cells

To perform the multiplex PCR allowing the amplification of the full HPV16 genome, we prepared two pools of primers 1A and 2A (Fig. 1) comprising non-overlapping primer pairs in equivalent quantities (each primer at a concentration of 7.14 µM), namely, #1, #3, #5, #7, #9, #11, #13 and #2, #4, #6, #8, #10, #12, #14, and two pools, 1B and 2B, where the quantity of primer pairs that had shown low amplification in previous tests was doubled. Thus, the quantity of primer pairs #13 in pool 1B and #8 in pool 2B was doubled (each primer at 6.25 µM). Then, the amplification was performed on several amounts of the SiHa cell line (5, 10, 12.5, and 15 ng) by adapting the primer volume to obtain a final concentration of each primer at 0.2 µM, except 0.4 µM for primer pairs whose volume was doubled (0.35 µL for 1A and 2A; 0.4 µL for 1B and 2B). Gel analysis showed the presence of a 700 bp band with different quantities of DNA extracts tested and in a comparable manner (Fig. S4).

**Fig 3:**
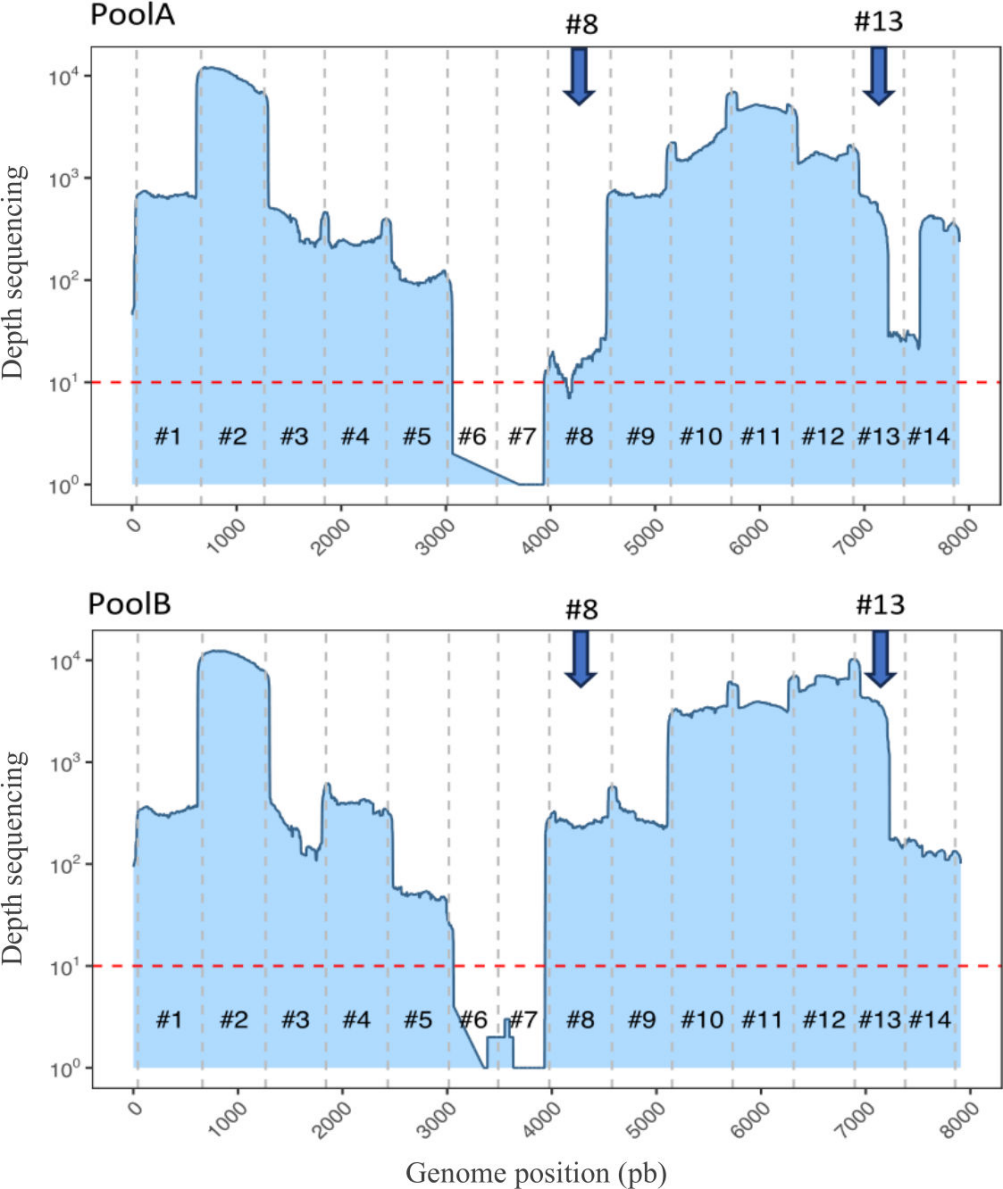
Coverage of the HPV16 genome after a multiplex PCR approach combined with ONT sequencing on the SiHa cell line at 12.5 ng with Pool A (primers in equivalent concentration) or B (primers #13 and #8 in double quantities). The red dotted line represents a threshold sequencing depth at 10×, and the vertical black dotted lines separate the 14 fragments amplified by the multiplex PCR.

The ONT sequencing results confirmed that fragments #6 and #7 were not amplified at the HPV genome integration region in the SiHa cell line, resulting in zero sequence coverage in this region. Furthermore, coverage at primer pairs #8 and #13 was significantly improved when the corresponding primer concentrations were doubled in pool B (Fig. 3).

### Evaluation of the multiplex PCR approach

#### Specificity

First, to confirm the reliability of the multiplex PCR approach, we compared the consensus sequences of the SiHa cell line, P37, and P89 obtained with the Sanger and ONT sequencing. The similarity of the sequences for the three samples was 99.68, 99.97, and 99.85%, respectively (data not shown).

We then amplified 12 extracted DNA tested positive for hrHPV (other than HPV16) with various genotypes and Ct values (Table S3). Gel analysis showed that only one extract out of 12 (patient A11) was amplified with pools 1B and 2B, whereas this patient had a co-infection involving HPV16 and HPV45. For the other extracts, only slight smears were observed, as well as a smaller band (<500 bp) for patient A3 with pool 1B (Fig. S5). Subsequent ONT sequencing showed 100% similarity to HPV16 for patient A11 without read mapping on the HPV45 genome and failed for all the other samples.

#### Sensitivity

We then tested the sensitivity of the multiplex PCR approach with pools 1B and 2B on six semi-quantified positive HPV16 samples (including 2 +, 2 ++, and 2 +++). Agarose gel analysis showed that samples +++ were correctly amplified (2/2 amplified), while samples ++ were amplified in one case on two tested, and the samples + failed to be amplified (0/2 amplified) (Fig. S6). For further analysis, we focused on patients with samples HPV16 ++and HPV16 +++.

#### Application to patients

Finally, we performed the multiplex PCR approach on 70 samples, including 48 HPV16 ++and 22 HPV16 +++. All of them were tested with the Allplex HPV28 test. The median (IQR) Ct value was 22.9 (22.3–24.9) for HPV16 +++ and 30.6 (28.1–33.4) for HPV16 ++ (*P* < 0.0001).

**Fig 4:**
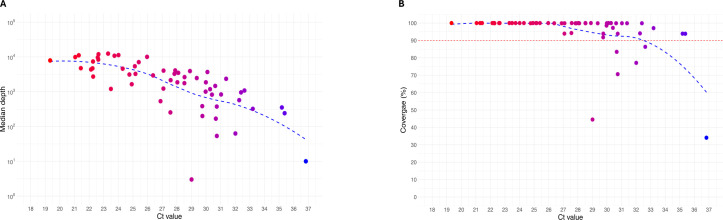
Results of the sequencing depth (**A**) and the HPV16 genome coverage (**B**) after the multiplex PCR approach combined with ONT sequencing according to the HPV16 Ct value determined with the Allplex HPV28 assay. The coverage was determined with a depth sequencing of 25*×.*

**Fig 5:**
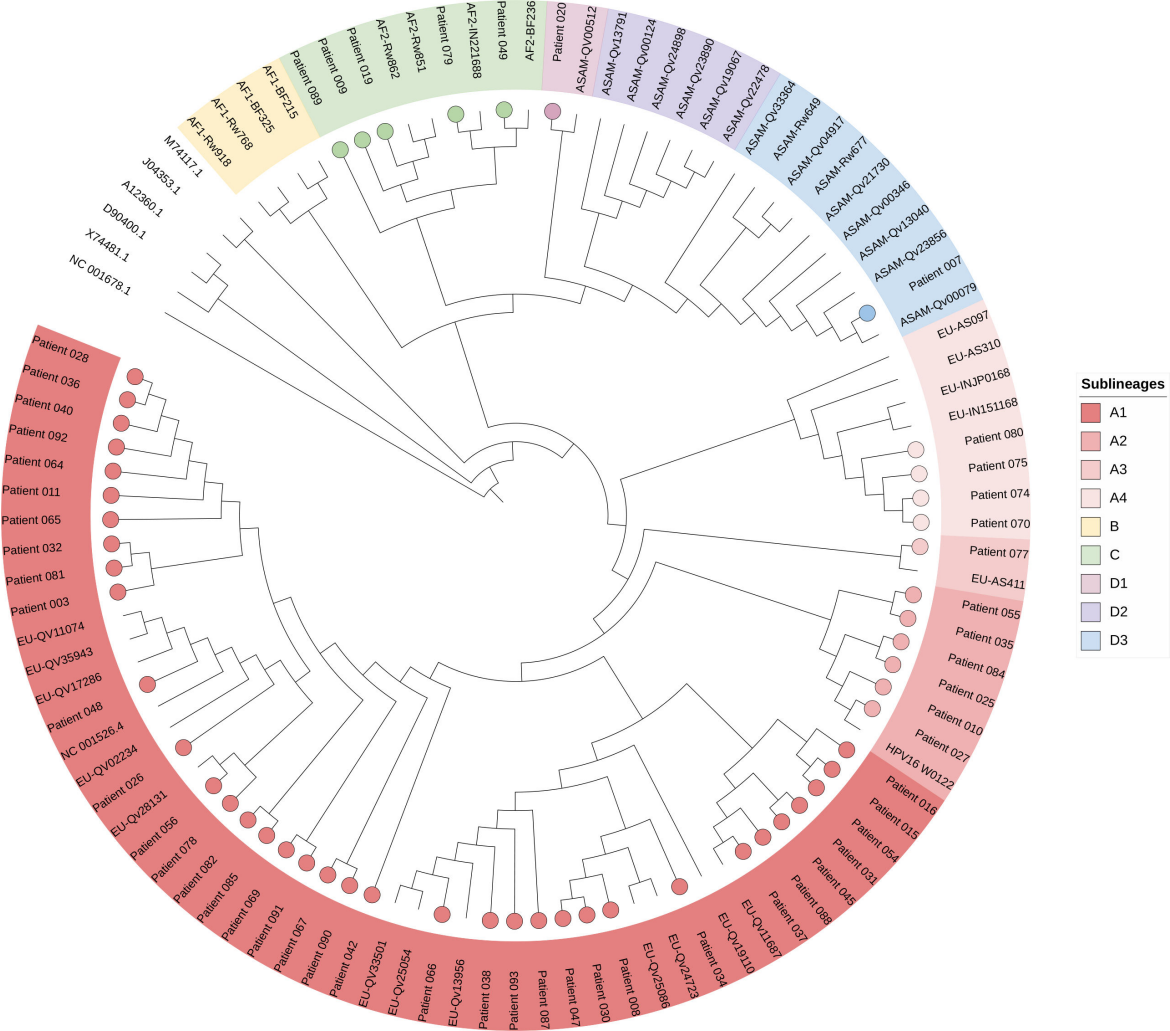
Maximum likelihood phylogenetic tree showing 54 full HPV16 genome sequences with the amplicon-based approach and Oxford Nanopore Technologies sequencing and 47 reference sequencing issued from GenBank and including lineages A, B, C, and D. Patients are highlighted with circles, and the different lineages/sublineages are represented by different colors*.* GenBank accession numbers: Patient #003: PV051119; Patient #007: PV051120; Patient #008: PV051121; Patient #010: PV051122; Patient #011: PV051123; Patient #015: PV051124; Patient #016: PV051125; Patient #019: PV051126; Patient #020: PV051127; Patient #025: PV051128; Patient #026: PV051129; Patient #027: PV051130; Patient #028: PV051131; Patient #030: PV051132; Patient #031: PV051133; Patient #032: PV051134; Patient #034: PV051135; Patient #035: PV051136; Patient #036: PV051137; Patient #037: PV051138; Patient #038: PV051139; Patient #040: PV051140; Patient #042: PV051141; Patient #045: PV051142; Patient #047: PV051143; Patient #048: PV051144; Patient #049: PV051145; Patient #054: PV051146; Patient #055: PV051147; Patient #056: PV051148; Patient #064: PV051149; Patient #065: PV051150; Patient #066: PV051151; Patient #067: PV051152; Patient #069: PV051153; Patient #070: PV051154; Patient #074: PV051155; Patient #077: PV051156; Patient #078: PV051157; Patient #079: PV051158; Patient #080: PV051159; Patient #081: PV051160; Patient #082: PV051161; Patient #084: PV051162; Patient #085: PV051163; Patient #087: PV051164; Patient #088: PV051165; Patient #090: PV051166; Patient #091: PV051167; and Patient #092: PV051168.

In total, 58 out of the 70 samples had effective ONT sequencing, and 12 samples failed to be amplified by the multiplex approach (median Ct value of 34.5 [32.8–36.9]). We generated a median of 82,889 (28,850–120,172) reads per sample. The median sequencing depth was 2,410 (761–4,703), and the median coverage at 25× was 99.9% (96.4–100). Between HPV16 +++ and HPV16 ++, the sequencing depth and the coverage were significantly different (*P* < 0.0001 for both). A good correlation was found between the HPV16 Ct value and the sequencing depth (*r* [confidence interval, CI 95%] = −0.79 [−0.87 to −0.66], *P* < 0.0001), as well as the coverage (*r* [CI 95%] = −0.73 [−0.83 to −0.58], *P* <0.0001) (Fig. 4A; Table S4). Below a Ct value of 27, all the genomes had a coverage above 99.9% (Fig. 4B; Table S4) and a sequencing depth above 100×. The sequences obtained showed that they belonged mainly to the A1 sub-lineage but also for some of them to the A2, A3, A4, C, D1, and D3 sub-lineages (Fig. 5).

## DISCUSSION

The important role played by HPV16 in the carcinogenesis of HPV lesions required an in-depth study of the genome genetic variability and the link to its transforming role. In that respect, we developed a multiplex PCR approach combined with ONT sequencing to characterize the HPV16 full genome.

The PCR step is essential to increase the sensitivity of the technique on patient samples and therefore the efficiency of sequencing HPV16 whole genome. The first step was to design the primer pools to cover the approximately 8,000 bp of the genome. To do this, PrimalScheme software was implemented with 41 reference genomes covering the four HPV16 (sub)lineages (A, B, C, and D) to ensure capturing the full diversity of this genotype (5). Initially, several fragment lengths were considered, from 300 to 1,000 bp, in order to find the best compromise between a correct overlap and good sensitivity of the PCR (16). Obtaining 700 bp fragments seemed to be the best option, and for this, 14 primer pairs were designed. Finally, to cover the entire genome in its episomal form, the primer pairs #1 and #14 present at the ends of the genome were designed to obtain a complete sequence of the HPV16 genome.

All primer pairs were tested on the SiHa cell line, and only the two fragments #6 and #7 targeting the E2 gene could not be validated. However, this result was not related to a lack of sensitivity or robustness since these primer pairs were subsequently successfully validated on samples from patients infected with HPV16. This led us to consider that the E2 gene was disrupted or deleted following its integration into the cellular genome, as already reported in the literature (17). Indeed, the integration of the viral genome after disruption of the E2 gene deregulated and increased the expression of the viral oncogenes E6 and E7 and thus promoted carcinogenesis (18, 19).

When setting up the multiplex PCR, results obtained in monoplex PCR were used to improve pools 1 and 2 to overcome some amplification weaknesses for primers targeting the E5–L2 (#8) and L1–URR (#13) regions. By doubling the primers concerned, a very good coverage across the entire genome was obtained (16).

The design was based specifically on HPV16 sequences, but it was important to determine their specificity, as in clinical practice, multi-infection with hrHPV is frequently found (20). Twelve samples infected or co-infected with a wide variety of hrHPV genotypes and often involved in HPV-carcinogenesis (i.e., HPV 18, 31, 33, 35, 39, 45, 51, 52, 56, and 58) were tested. Importantly, none of these genotypes could be amplified or sequenced, thus demonstrating the specificity of the primers with respect to HPV16. This specificity was reinforced by the study of a sample co-infected with HPV45 and HPV16, which made it possible to obtain 100% HPV16 genome without any reads related to HPV45. Furthermore, the fact that patient sequences could be identified as being related to different sublineages shows that the primers have the capacity to amplify different sequences within the HPV16 genotype.

Following a sensitivity test of the multiplex PCR approach, it was observed that samples with a Ct value greater than 40 could not be amplified. On the contrary, for samples with a Ct value between 30 and 40, the amplification was random. When the amplification was successful, the sequence coverage was variable and limited. To overcome this lack of amplification, a new design using smaller fragments (i.e., 300 or 400 bp instead of 700 bp) could be tested. However, the design of the primers on PrimalScheme software shows a weakness when the fragments are small, with some fragments that have little or no overlap, which can lead to gaps in the resulting sequence. For these samples, an alternative approach using specific capture probes could be considered. However, it would require the development of robust bioinformatics tools and would significantly increase the cost (21). Interestingly, all the samples with a Ct value below 27 had a nearly 100% coverage with a sequencing depth higher than 100×. From our clinical practice, these results are similar to those obtained for SARS-CoV-2 and VRS whole-genome sequencing and based on the same multiplex PCR and ONT sequencing approaches (22).

In conclusion, we set up a multiplex PCR approach combined with ONT sequencing to characterize the full genome and diversity of the HPV16 (sub)lineages. This approach shows good sensibility and specificity with limited cost, opening new perspectives in the field of whole-genome HPV and viral sequencing.

## Data Availability

The data that support the findings of this study are available from the corresponding author upon reasonable request. The nucleotide sequences of the HPV16 genome have been submitted and are available on GenBank: PV051119; PV051120; PV051121; PV051122; PV051123; PV051124; PV051125; PV051126; PV051127; PV051128; PV051129; PV051130; PV051131; PV051132; PV051133; PV051134; PV051135; PV051136; PV051137; PV051138; PV051139; PV051140; PPV051141; PV051142; PV051143; PV051144; PV051145; PV051146; PV051147; PV051148; PV051149; PV051150; PV051151; PV051152; PV051153; PV051154; PV051155; PV051156; PV051157; PV051158; PV051159; PV051160; PV051161; PV051162; PV051163; PV051164; PV051165; PV051166; PV051167; and PV051168.

## SUPPLEMENTAL MATERIAL

Supplemental tables and figures spectrum.03073-24-s0001.xlsx Table S1 to S4 and Fig. S1 to S6.

ASM does not own the copyrights to Supplemental Material that may be linked to, or accessed through, an article. The authors have granted ASM a non-exclusive, world-wide license to publish the Supplemental Material files. Please contact the corresponding author directly for reuse.
